# Environmental DNA revealed the fish community of Hokkaido Island, Japan, after invasion by rainbow trout

**DOI:** 10.3897/BDJ.8.e56876

**Published:** 2020-10-29

**Authors:** Akio Imamura, Kana Hayami, Masayuki K. Sakata, Toshifumi Minamoto

**Affiliations:** 1 Hokkaido University of Education, Hokkaido, Japan Hokkaido University of Education Hokkaido Japan; 2 Kobe University, Kobe, Japan Kobe University Kobe Japan

**Keywords:** biological invasion, competitive exclusion principle, metabarcoding, native salmonids, seasonal change

## Abstract

In freshwater ecosystems, invasive salmonid fishes can have a significant impact on native fish species. Detecting the invasion and its negative effects is critical for the conservation of native fish communities. We examined the species composition and seasonal changes in the freshwater fish community, including salmonids, on the Kamikawa Plain, Hokkaido Island, Japan, using environmental DNA (eDNA) metabarcoding. We detected 23 fish species in 176 samples collected from 16 sites over 12 months (October 2018 – August 2019). Between 11 and 20 species were detected at each site, including five native salmonids (*Oncorhynchus masou*, *Oncorhynchus keta*, *Parahucho perryi*, *Salvelinus leucomaenis leucomaenis* and *Salvelinus malma krascheninnikova*). The invasive alien rainbow trout *Oncorhynchus mykiss* was detected at all 16 sites and it was the most commonly detected salmonid. Although we found no obvious competitive exclusion of native salmonids by rainbow trout in the study area, the invasive species occurred more often and at more sites than any of the natives. We also determined the occurrence and seasonal changes in the fish community, classified as native salmonids, invasive rainbow trout, Cypriniformes and other benthic fishes. There were fewer species overall in winter, but the sites with higher species richness in winter were on the lower reaches of the river. In addition, we detected domestic invaders, such as the topmouth gudgeon, *Pseudorasbora parva*, although they were less prevalent than rainbow trout. These results show the effectiveness of eDNA metabarcoding, which can be used for surveying species richness at an ecosystem scale. In particular, the detection of the early stages of establishment and spread of invasive species can be achieved by eDNA monitoring.

## Introduction

There is a high species richness (seven species and two subspecies) of native salmonid fish on Hokkaido Island in the Japanese Archipelago, as *Parahucho perryi* (Brevoort 1856) (Sakhalin taimen), *Salvelinus leucomaenis leucomaenis* (Pallas 1814) (white-spotted char), *Salvelinus malma krascheninnikova* Taranetz 1933 (Dolly Varden char), *Salvelinus malma miyabei* Oshima 1938, *Oncorhynchus masou* (Brevoort 1856) (masu salmon), *Oncorhynchus keta* (Walbaum 1792) (chum salmon), *Oncorhynchus gorbuscha* (Walbaum 1792) (humpback salmon), *Oncorhynchus nerka* (Walbaum 1792) (sockeye salmon) and *Oncorhynchus tshawytscha* (Walbaum 1792) (king salmon). In Japan, the translocation of freshwater fishes, including salmonids, both within the country and from overseas, began in the 1800s (Taniguchi et al. 2000). Since then, wild populations of introduced salmonids have been established throughout Japan (Taniguchi et al. 2000). In Hokkaido Island, the exotic invaders *Oncorhynchus mykiss* (Walbaum 1792) (rainbow trout) and *Salmo trutta* Linnaeus 1758 (brown trout) have had negative effects on native species, including through antagonistic relationships (i.e. predation) and competitive exclusion (Baxter 2007, Taniguchi et al. 2000, Sahashi and Morita 2016, Hasegawa et al. 2004, Hasegawa and Maekawa 2006). For example, Morita et al. (2004) reported that invasive species caused displacement of native species and Hasegawa and Maekawa (2006) reported that invasive species increased the competition between two native salmonid species. It is clear that invasive salmonid fishes have a significant impact on major native fish species in the freshwater ecosystems of Hokkaido Island.

Salmonid species, such as salmon, trout and charr, generally need different habitats, based on the season of the year and stage of their life cycle (Bjornn and Reiser 1991). As many salmonid species follow either anadromous migration and spawning patterns as they mature, habitat fragmentation can lead to a loss of connections amongst meta-populations and thus increase the risk of local extinction (Morita et al. 2009). In addition, the invasion of a closely-related species, such as rainbow trout, might increase the risk of competitive exclusion.

Freshwater ecosystems on Hokkaido Island have been invaded by other non-native fish species as well. For example, *Silrus asotus* Linnaeus 1758 (Far Eastern catfish) was found in the middle reaches of the Ishikari River in Hokkaido (Kobayasi and Adachi 1957). *Channa argus* (Cantor 1842) (northern snakehead) also has been found in parts of Hokkaido, including the capital, Sapporo City (Katayama et al. 2018). The full spatial distribution of these invasive species is not known. In addition, there are concerns about the spread of invasive cyprinid species from other regions of Japan. For example, *Pseudorasbora parva* (Temminck & Schlegel 1846) (topmouth gudgeon), an endemic Japanese gobionid fish, is known as the “Asian killer fish” in Europe. It has been established in Europe and its parasitic pathogens (*Sphaerothecum destruens* Arkush et al. 2003, the rosetta agent) have caused a drastic decrease in native European cyprinid species (Gozlan et al. 2005). This species has expanded its habitat in north-eastern Japan, including Hokkaido Island (Hikita 1964, Sugano and Kanaiwa 2016, Sugano et al. 2019). Thus, detection of the invasive species and their negative effects is critically important for the conservation of native fish communities (Baxter 2007, Hasegawa and Maekawa 2006, Hasegawa et al. 2004, Morita et al. 2004, Sahashi and Morita 2016, Taniguchi et al. 2000).

In Japan, although the rainbow trout is designated as an invasive alien species that requires careful management (Ministry of the Environment, Japan 2015, in Japanese), Hokkaido Prefecture postponed the designation and the prohibition against stocking the species in 2015 to promote fisheries and recreational fishing. This decision may well underestimate the negative effects of rainbow trout on the salmonid-rich ecosystems of Hokkaido Island. To assess the implications of the decision, it is necessary to identify the invasion status and the negative effects that rainbow trout can have throughout the year at both the fish community scale and the regional scale.

Environmental DNA (eDNA) metabarcoding allows sequences found in eDNA to be associated with a taxonomic name. This technique is a powerful molecular tool for surveying species richness non-invasively, which works in many ecosystems (Deiner et al. 2017). Nakagawa et al. (2018) verified the validity of eDNA metabarcoding by comparing it with a traditional survey in rivers of Japanese Honshu Island. Keskin et al. (2016) used it to construct a species inventory including both rare domestic species and invasive species in the lake of Turkey. Yamamoto et al. (2017) and Thomsen et al. (2012) described marine fish communities using eDNA metabarcoding, while Stoeckle et al. (2017) used eDNA metabarcoding to survey seasonal fish abundance. Thus, eDNA metabarcoding should be sufficient to detect the negative effects of invasive fish species on native fish communities. Although some studies reported eDNA metabarcoding throughout the year (Bista et al. 2017, Hänfling et al. 2016), there have been few studies using continuous eDNA sampling throughout the year to test the negative impacts of invasive fish species.

The Kamikawa Plain, Hokkaido, contains Daisetsuzan National Park, the largest national park in Japan; the Ishikari River, the largest river in Hokkaido, originates in the Daisetsu Mountains. Thus, conservation of the fish community in this area is important for freshwater ecosystems in Hokkaido. We investigated the species composition and seasonal changes in the freshwater fish community, including salmonids, on the Kamikawa Plain using eDNA metabarcoding. We executed monthly water sampling, including heavy snow conditions in midwinter, using methods developed in our previous studies (Imamura et al. 2019, Minamoto et al. 2019) to assess the fish communities and detect invasive species in Hokkaido.

## Material and Methods

### Site Selection, Water Sampling and Filtration

We selected 16 sites along the Ishikari River, its tributary including the Chubetsu River, the Masutori-gawa River and the Antaroma River, in the Kamikawa Plain, Central Hokkaido (43.6 – 43.9 N long., 142.2 – 142.7 E lat., 100 – 450 m alt., Suppl. material 1, Fig. 1). These include six sites around the 13-year-old Chubetsu Reservoir (Sites J-O in Fig. 1). Masu salmon have been observed migrating up the Masutori-gawa River for reproduction (Site B in Fig. 1, personal observation) and chum salmon have been observed in the Ishikari River and Chubetsu River (Sites F and G, respectively, in Fig. 1, personal observation).

Water sampling was conducted once a month from October 2018 to August 2019. We collected a single 900-ml surface water sample per site using a plastic bottle. For midwinter sampling, we followed the methods for water sampling during the freezing season established by Minamoto et al. (2019) and Imamura et al. (2019). These methods included drilling a hole, if needed, to sample the surface water. To prevent DNA degradation, 1 ml of 10% benzalkonium chloride solution (Takeda Pharmaceutical Company Ltd., Tokyo) was added to each sample (Yamanaka et al. 2017) and the water samples were filtered on the sampling day using 47-mm glass fibre filters with a nominal pore size of 0.7 μm (GF/F, GE Healthcare, Chicago, IL, USA). A single 900-ml sample of pure water was similarly filtered on the sampling day as a filtration blank. Filter papers were stored at -25°C until DNA extraction. All equipment used in water collection and water filtration was bleached using a commercial bleach (Kao Haiter, < 6% sodium hypochlorite solution, Kao Corporation, Tokyo) and diluted sodium hypochlorite solution of > 0.1% before use to prevent contamination (The eDNA Society 2019). Disposable gloves were used during all procedures to minimise the risk of contamination. DNA extraction from filters was conducted according to Minamoto et al. (2019). Briefly, each filter was transferred to a Salivette tube (Sarstedt, Nümbrecht, Germany) and 400 μl of Buffer AL (Qiagen, Hilden, Germany) and 40 μl of Proteinase K (Qiagen) were added. After incubation at 56°C for 30 min, the tubes were centrifuged at 3,000×g for 3 min. Then, 300 μl of Tris-EDTA (TE) buffer was added to the filters and the tubes were re-centrifuged at 3,000×g for 1 min. DNA was extracted from the collected elution using a DNeasy Blood & Tissue Kit (Qiagen) according to the manufacturer’s protocol. The final DNA samples (100 μl) were stored at -25°C until subsequent molecular biological assays.

To assess the fish diversity at each site, eDNA metabarcoding, targeting the mitochondrial 12S rRNA gene, was performed using MiFish-U primers (Miya et al. 2015). Each first polymerase chain reaction (PCR) reaction contained 6.0 μl of 2×KAPA HiFi HotStart ReadyMix (KAPA Biosystems, Wilmington, MA, USA), 300 nM of each of the primers (forward: 5’- ACACTCTTTCCCTACACGACGCTCTTCCGATCTNNNNNNGTCGGTAAAACTCGTGCCAGC-3’, reverse: 5’- GTGACTGGAGTTCAGACGTGTGCTCTTCCGATCTNNNNNNCATAGTGGGGTATCTAATCCCAGTTTG-3’) and 1.0 μl of the template eDNA. The thermal conditions of the first PCR consisted of an initial 3 min denaturation at 95°C; 40 cycles of 98°C for 20 s, 65°C for 15 s and 72°C for 15 s; and a final step of 72°C for 5 min. There were four technical replicates for each sample and ultrapure water was used instead of an eDNA sample as a PCR blank. After the first PCR, the four replicates were pooled and purified using the SPRIselect Reagent Kit (Beckman Coulter, Brea, CA, USA), according to the manufacturer’s instructions. DNA concentrations of the purified products were quantified with a Qubit dsDNA HS assay kit and a Qubit fluorometer 3.0 (Thermo Fisher Scientific, Waltham, MA, USA) and the PCR products were diluted to 0.1 ng/μl before being used as templates for the second PCR.

The second PCR was performed to add adapter sequences for high-throughput sequencing and 8-bp index sequences. Each second PCR reaction contained 6.0 μl of 2×KAPA HiFi HotStart ReadyMix, 300 nM each of forward and reverse primers, 1 μl DNA template and 1 μl ultrapure water. The final volume was 12 μl. The thermal conditions of the second PCR consisted of an initial 3 min denaturation at 95°C, 12 cycles of 98°C for 20 s and 72°C for 30 s, followed by 72°C for 5 min. All of the second PCR amplicons were pooled and diluted five times with ultrapure water. A 200-μl sample of the size-selected library sample was obtained using E-Gel SizeSelect 2% (Thermo Fisher Scientific) with the E-Gel Precast Agarose Electrophoresis System (Thermo Fisher Scientific). The size of the library sample was confirmed using an Agilent 2100 Bioanalyzer (Agilent Technologies, Santa Clara, CA, USA). This process was performed at Environmental Research and Solutions Co. Ltd. (Kyoto, Japan) for the samples collected in October and November 2018 and at Kobe University (Kobe, Japan) for the rest of the samples. The library sample was sequenced using an Illumina MiSeq or iSeq with 2 × 150 bp pair-end kits (Illumina, San Diego, CA, USA).

Raw reads from a high-throughput sequencer were preprocessed and analysed using USEARCH v10.0.240 (Edgar 2010). The criteria for data preprocessing and analysis followed those of Sakata et al. (2020). The details are as follows: forward and reverse reads (reads 1 and 2) were merged and low-quality (Phred score < 2) tails were trimmed. Both primer sequences were removed from the assembled reads. To remove reads with an expected error rate (Edgar and Flyvbjerg 2015) of > 1% and too-short reads of < 100 bp, quality filtering was performed. Identical reads (the same length and same sequence) were clustered. To generate amplicon sequence variants (ASVs), clustered reads were denoised using the “unoise” algorithm. Finally, ASVs were identified using an online basic local alignment search tool (Nucleotide collection of Standard database of BLAST, https://blast.ncbi.nlm.nih.gov/Blast.cgi), based on a 98.5% homology criterion (two nucleotide differences allowed).

To scrutinise the results obtained, we performed the following three steps. First, the number of species reads detected in the negative controls (i.e. filtration blanks or PCR blanks) was subtracted from the corresponding samples. Second, pure marine fish species were excluded as contamination because there were no marine fish in the studied area. Finally, every species detected in only one sample was regarded as a potential false positive and so these species were excluded from the subsequent analysis. We converted the read counts to presence/absence data and used these in further analyses.

### Statistical Analysis

All statistical analyses, including mapping, were conducted using R 3.5.2 (R Core Team 2018 https://www.R-project.org). Geographical information about the sites was collected using Google Maps (geodetic system: WGS84). The great-circle distance between the sites was calculated using the function spDistsN1 of the “sp” package. The “maptools” and “rgdals” packages were used to draw the results on the map. We used the “tidyverse” package to tidy up the data and we used the “vegan” package to examine the fish species composition and its spatial autocorrelation. The spatial autocorrelation was calculated using the mantel function of “vegan” with the distance matrix with the Jaccard Index because we collected presence/absence data. Mapping was conducted with geographical data from the Ministry of Land, Infrastructure, Transport and Tourism of Japan (http://nlftp.mlit.go.jp/ksj/index.html). The “ggplot2” and “gplot” packages were used to draw graphs, including a heatmap.

The factors affecting the fish community at each site on each sampling date were analysed with permutational multivariate analysis of variance (PerMANOVA) using the adonis function of “vegan”, with site, sampling date and presence of rainbow trout as explanatory variables. In PerMANOVA, sites were classified into three categories: mainstream (Sites A, D, E, F, H, I and J), mid-sized stream (Sites C, G, M, O and P) and small tributaries (Sites B, K, L and N). The sampling dates were categorised for four seasons, according to the Japanese seasonal categories and the degree of snowfall in Hokkaido, as follows: autumn (October and November 2018), winter (December 2018 and January, February and March 2019), spring (April and May 2019) and summer (June, July and August 2019).

Finally, to determine whether there was a competitive exclusion relationship between each major fish species and *O. mykiss*, a generalised linear model (GLM), using a binomial distribution (link function was 'logit') with the explanatory variables as site, sampling date and presence of rainbow trout, was run. Model selection was performed using Akaike's Information Criterion involving the function stepAIC of package 'MASS'.

## Results

### DNA Detection by eDNA Metabarcoding

We successfully detected DNA of 25 fish species from 10 families, including Cyprinidae and Salmonidae (Suppl. material 1). On average, the number of DNA reads per sample was 40,647.1 ± 7,810.0 (mean ± S.D.). The number of species reads detected in the negative controls (i.e. filtration blanks or PCR blanks) are presented in Suppl. material 2. False positives in the negative controls were 28 cumulative species amongst 550 cumulative species (550 = 22*25 species, i.e. filtration blanks and PCR blanks of 11 research date*25 species). False positives per sampling date ranged from 0 to 4 species. The reads, identified as marine fish species, had the same specific sequences as the marine fish species.

The number of species per site ranged from 11 to 20 (Fig. 1 and Suppl. material 1). The DNA from Ezo-ugui *Pseudaspius sachalinensis* (Nikolskii 1889) (formerly the former genus *Tribolodon* is considered a synonym of Pseudaspius, according to Sakai et al. 2020) of Leuciscidae, *Barbatula barbatula* (Linnaeus 1758) (stone loach) of Nemacheilidae, *Misgurnus anguillicaudatus* (Cantor 1842) (weather loach) of Cobitidae and *Cottus nozawae* Snyder 1911 (wrinklehead sculpin) of Cottidae were detected at all 16 sites, as was the DNA of the invasive rainbow trout.

We detected native salmonids *Parahucho perryi* (Sakhalin taimen), *Salvelinus leucomaenis leucomaenis* (Pallas 1814) (white-spotted charr), *Salvelinus malma krascheninnikova* (Dolly Varden char), *Oncorhynchus masou* (masu salmon) and *O. keta* (chum salmon). The ASVs of these salmonids, except *P. perryi*, did not identify the fish to the species level though eDNA metabarcoding because there are closely-related subspecies or other species with which they can be misidentified. For example, there are a few char subspecies in Japan’s Honshu Island that are very similar to *S. leucomaenis leucomaenis* (white-spotted charr). Other species that have similar DNA are *Oncorhynchus masou* Brevoort 1856 (satsukimasu trout) and *O. masou* (masu salmon); *Salvelinus fontinalis* (Mitchill 1814) (brook trout) and *S. malma krascheninnikova* (Dolly Varden charr); and humpback salmon and chum salmon. Author AI directly identified the species present here through specimen capture, as shown in Suppl. material 1 (see Minamoto et al. (2019) and Imamura et al. (2019) for details). Thus, the ASVs were treated as the species named here. We did not detect DNA from any invasive salmonid except for rainbow trout.

We detected many species of the Cyprinidae and closely-related families, including *Cyprinus carpio* Linnaeus 1758 (common carp), *Carassius cuvieri* Temminck & Schlegel 1846 (Japanese crucian carp), *Pseudorasbora parva* (topmouth gudgeon), *Gnathopogon caerulescens* (Sauvage 1883) (hon-moroko), *Tanakia lanceolata* (Temminck & Schlegel 1846) (slender bitterling), *Rhynchocypris lagowskii* (Dybowski 1869) (Amur minnow), *Pseudaspius hakonensis* (Günther 1877) (Japanese dace) [the former genus *Tribolodon* Sauvage 1883 is currently considered a synonym of *Pseudaspius* Pallas 1776, according to Sakai et al. (2020)], which are categorised as domestic invaders from within the Japanese Archipelago (Hikita 1964, Katayama et al. 2018, Kobayasi and Adachi 1957, Mabuchi et al. 2005, Sugano and Kanaiwa 2016, Sugano et al. 2019, Takami and Kawamura 2008). The native Cypriniformes species *Rhynchocypris percnurus* (Pallas 1814) (swamp minnow) was also detected (Suppl. material 1).

We also detected native sand lamprey *Lethenteron* sp. of Petromyzontidae, the exotic invader *Paramisgurnus dabryanus* Dabry de Thiersant 1872 (pond loach) of Cobitidae and domestic invader *Silurus asotus* Linnaeus 1758 (Far Eastern catfish) of Siluridae, from some sites on a few sampling dates.

### DNA Detection per Species

The number of samples of each salmonid and other frequently-detected species (Suppl. material 3), with possible false positives excluded, that were found at each site is illustrated in Fig. 2 and Suppl. material 4. *Barbatula barbatula* (Stone loach) DNA was detected at all 16 sites throughout the study (165 of 176 samples), making it the most common ASV. *Cottus nozawae* (wrinklehead sculpin) DNA was also detected at all sites throughout the year (113 samples), although there was a lower rate of detection at near-reservoir sites. DNA from *Pseudaspius sachalinensis* (Ezo-ugui), *Pseudaspius hakonensis* (Japanese dace) and *Misgurnus anguillicaudatus* (weather loach) was detected in 82, 48,and 66 samples, respectively, with higher rates at the lower-reach sites and lower rates at the upper reaches and smaller tributaries.

*Oncorhynchus mykiss* (rainbow trout) was detected at all sites throughout the research (105 samples), making it the most abundant salmonid and it was detected repeatedly at the near-reservoir sites. Although *O. masou* (masu salmon) DNA was detected in almost all (15 of 16) sites, there was a total of just 72 samples, with fewer samples from the near-reservoir sites, which is a pattern different from that of the *O. mykiss* (Suppl. material 3). *S. leucomaenis leucomaenis* (white-spotted charr) DNA was detected at 12 sites, in 32 total samples. *S. malma krascheninnikova* (Dolly Varden charr) DNA was detected at 11 sites and in more samples in the upper sites and around-dam sites than elsewhere (45 samples in total). DNA of chum salmon was detected at 11 sites and in 18 samples in total, although the detection from the above-reservoir site was not reliable because no stock has been reported there. DNA of critically-endangered species Sakhalin taimen, which has not been captured around our research sites since the 1970s (Takami and Kawamura 2008), were detected at 2 sites (2 samples in total)

### Native Salmonids and Invasive Rainbow Trout

We examined the relationship between the native and invasive salmonids and whether there was evidence of competitive exclusion, by dividing all the species into four groups: invasive *O. mykiss* (rainbow trout), native salmonids, Cypriniformes and others, drawing species-count graphs for each site by research date (Fig. 3). *Oncorhynchus mykiss* DNA was detected in 105 of the 176 water samples, making this the most commonly-detected salmonid. However, the relationship between the native and invasive salmonids was not one of exclusion, as there were few water samples in which *O. mykiss* was the only salmonid detected (Fig. 3). The samples that did not contain DNA from *O. mykiss* contained fewer total species. This tendency was remarkable at the sites around the Chubetsu Reservoir (Fig. 2J–O).

As for the seasonal change in the species number per site, sites A, C, D, F and G contained more species (7–11) in winter, although the winter samples from other sites tended to contain fewer species (2–5). These sites are at the lower reaches of our research rivers (Fig. 3). Higher species richness at these sites resulted from the presence of more cyprinid species (ca. 5) and those from other families. The sites at the upper reaches and smaller tributary (P and B, respectively, in Fig. 3) contained fewer species than those of other sites.

### Species Composition and Similarity

We drew a heatmap based on the Jaccard Similarity Index of the species composition pooled for yearly data for each site (Fig. 4). The heatmap denoted three clusters. Sites B and N are small tributaries and clustered as neighbours and sites D and J were clustered nearby. The sites around the Reservoir and other mainstream, mid-size stream and small tributary sites were mixed in the remaining two clusters. We tested the spatial autocorrelation with the R function mantel with 10,000 permutations and the matrix using the Jaccard Similarity Index. These tests showed a low correlation (Mantel statistic r = -0.006, Significance = 0.51).

We analysed the effects of rainbow trout on the fish community using PerMANOVA and GLM. The PerMANOVA showed that all variables of site, sampling date and rainbow trout had significant effects (R^2^ = 0.036, Suppl. material 5). However, further analysis using GLM found that the effects of rainbow trout were not significant or significantly positive on any of the major fishes (see Suppl. material 6), including masu salmon, Dolly Varden charr, white-spotted charr, stone loach, wrinklehead sculpin, pond loach and Ezo-ugui.

## Discussion

### Detection of Salmonid Species

We successfully detected the fish community composition using eDNA metabarcoding. Our research targeted the freshwater ecosystems in Hokkaido, Japan, where the invasive salmonid, *O. mykiss* (rainbow trout), have been established and is considered to negatively affect native salmonids and other fish. As *O. mykiss* DNA was detected at all sites and showed the highest frequency (105 samples) of all the salmonids, it seems that the species could be the dominant salmonid around the Kamikawa Plain. Around the Chubetsu Reservoir, salmonids, other than the rainbow trout, were detected at fewer sites and lower frequency than that of *O. mykiss*. This is because the species is thought to have been artificially stocked into the Reservoir for recreational fishing.

Amongst the native salmonids, *O. masou* (masu salmon) DNA was detected at 15 of the 16 sites. It was observed that at Site A, *O. masou* up-migrated into the Masutori-gawa River to spawn in autumn, although larger rainbow trout individuals inhabited this area sympatrically along with the masu salmon. This species is not considered to be endangered, although continuous and long-term assessments are needed, as earlier studies have found that the rainbow trout is a potential threat to native salmonids (Morita and Yokota 2002, Sahashi and Morita 2016).

*Salvelinus malma krascheninnikova* (Dolly Varden charr) was detected at just 11 sites, this species tending to coexist with rainbow trout in the upper reaches, however, as previously reported by Imamura et al. 2019. Although the species was denoted as vulnerable (VU) in the Japanese Red List (Ministry of environment, Japan 2019), it is not considered to be endangered in our research rivers because it was found at a reasonably high number of sites and total samples (11 sites and 45 samples in total). Rainbow trout DNA was detected in 38 of the 45 *S. malma krascheninnikova*-positive samples. This result and our GLM, support the report of Imamura et al. (2019), who found that there is co-existence of species which suggests no competitive exclusion between *S. malma krascheninnikova* and *O. mykiss*.

*Salvelinus leucomaenis leucomaenis* (white-spotted charr) was detected in 32 samples. Although the species has not been denoted in the Japanese Red List, we believe there is a future threat for this species on the Kamikawa Plain. *Oncorhynchus mykiss* DNA was detected in 25 of these 32 samples; however, Imamura et al. (2019) did not reject competitive exclusion between *S. leucomaenis leucomaenis* and *O. mykiss*. Since *S. leucomaenis leucomaenis* tends to migrate seasonally (i.e. sea-run and up-migration), they are the native salmonid most negatively affected by artificial structures, such as dams (Morita et al. 2009). Their life history is different from that of the *S. malma krascheninnikova* of the Kamikawa Plain, which does not migrate to the sea.

The DNA of *O. keta* (chum salmon) was detected 18 times across 11 sites. It was detected four times near the Chubetsu Reservoir, at one below-reservoir site (Site J in December 2018) and three above-reservoir sites (Sites K, M and O in August 2018). While the species has not been reported to up-migrate to Site J, author AI observed them spawning at the lower site (Site G). The other sites are above the Reservoir and the species cannot pass the dam structure. It is possible that the DNA found at the above-reservoir sites was derived from the eggs of the salmon, which are commonly used for recreational fishing. Since the Chubetsu Reservoir is a large barrier of more than 80 m height, there is no possibility of the transportation of the carcass by wild animals.

We detected DNA from *P. perryi* (Sakhalin taimen), which is denoted as critically endangered (CR) in the Japanese Red List, in two samples from two sites. Takami and Kawamura (2008) reported that the species has not been captured from the Ishikari River on the Kamikawa Plain since the 1970s. One possible explanation for our finding is that it is a re-discovery or recovery of the locally extinct species and another is that it is the result of artificial stocking for recreational fishing because some anglers have posted comments to social networking services about personally re-stocking the species. One way to determine this would be to survey the species using a specific eDNA protocol (Mizumoto et al. 2017).

### Comparison between Sites and Seasons

In all, we detected the DNA of 25 fish species by metabarcoding eDNA in water samples. We examined the differences in species composition amongst the research sites and the seasonal changes in this composition. The low spatial autocorrelation of the species composition and the heatmap analysis revealed that the composition at each site was determined by multiple factors. We were able to detect DNA from samples taken in midwinter, which is the heavy snow season. The number of species tended to be lower in winter (December to March) than in other seasons. However, Sites A, C, D, F and G contained more species in winter than in summer, where they were located on the lower reaches of our sample rivers and could contain deeper pools, compared with the smaller, fast flowing tributaries found at sites in the upper reaches. This result suggests that they formed a winter refuge for the fish community. Although further studies are required on the quantitative effect and mechanisms of eDNA flow in lotic water (Shogren et al. 2017, Seymour et al. 2018), Handley et al. (2019) reported that the number of species changed temporally (summer and winter) and spatially (shore-offshore and depth in the lake) by eDNA metabarcording.

The reservoir sites had lower numbers of species, probably not because of competition with rainbow trout, but due to other unfavourable conditions or overwintering migration. While very little salmonid DNA was detected at reservoir sites in winter, *B. barbatula* (stone loach) and/or *M. anguillicaudatus* (weather loach) DNA was detected at those sites year-round. It is possible that the loach species do not migrate and thus overwinter around the Reservoir. At present, however, since we do not have detailed environmental data for the sites, we cannot fully explain the multiple factors influencing species composition.

### Cypriniformes as Domestic Invaders

We found the DNA of species, such as *C. carpio* (common carp), *Carassius* sp. (crucian carp), *T. lanceolata* (slender bitterling), *P. parva* (topmouth gudgeon), *G. caerulescens* (hon-moroko) and *S. asotus* (Far Eastern catfish), that are not native to Hokkaido Island. Their detailed distribution in Hokkaido was not identified in this study. Repeated detection of these species indicates that they should indeed be present in this area, if sampled using traditional methods. These species are thought to be domestic invaders from inside the Japanese Archipelago (Hikita 1964, Katayama et al. 2018, Mabuchi et al. 2005) and so more information is needed on when and where they are invading or have invaded. Unlike traditional surveys, eDNA analysis can be an effective way to discover new invasions of exotic or domestic origins. DNA from species, such as *T. lanceolata* (slender bitterling) and *G. caerulescens* (hon-moroko), were detected repeatedly in this study, although they were not previously reported to be present on Hokkaido Island. They are native to Japan’s Honshu Island and are thought to have been artificially introduced to Hokkaido for fisheries or recreational angling.

*Pseudorasbora parva* (topmouth gudgeon) is the most notable of the domestic and non-defined invaders found in this study and should receive the most attention. This species was detected at six sites (six samples in total), specifically four of the lower sites and two of the near-reservoir sites. This species has invaded many European water bodies and is colloquially called the "Asian killer fish" because its parasitic pathogen kills native Cypriniformes and might kill native salmonid fishes (Gozlan et al. 2005). It is possible that we can detect the eDNA of the pathogen and could evaluate the risk of invasion of *P. parva* in a future study.

As for other domestic invaders, we detected *C. carpio* (common carp), *C. cuvieri* (Japanese crucian carp) and *S. asotus* (Far Eastern catfish), which have established populations in Hokkaido Island (Kobayasi and Adachi 1957, Katayama et al. 2018). For example, although *S. asotus* has not previously been reported to inhabit the Kamikawa Plain, Kobayasi and Adachi (1957)reported that the species inhabit the Ishikari River, the 50-km lower reach of our research rivers. As these invaders were detected in a few samples and there have been few reports of capture, the relative importance or impact of this species on the native fish community around the Kamikawa Plain should be low at this time. Benthic fish species, such as *B. barbatula* (stone loach), *C. nozawae* (wrinklehead sculpin) and *M. anguillicaudatus* (weather loach), were detected at most sites and in most water samples. It is not clear whether *M. anguillicaudatus* (weather loach) is truly native to these sites or is a domestic invader. However, these benthic species are thought to be important elements of the fish community in Hokkaido, although they have not received much attention from fishery or commercial viewpoints.

### Conclusions and Caveats

In conclusion, we detected the distribution and seasonal changes of the fish community, including the native salmonids, the invasive *O. mykiss*, Cypriniformes and other benthic fish, such as *B. barbatula, C. nozawae* and *M. anguillicaudatus*. We also detected less-common domestic invaders. These findings used the technique of eDNA metabarcoding, which is capable of surveying species composition at the ecosystem scale. In particular, the detection of the early stages of establishment and spread of the invaders is best achieved by eDNA monitoring rather than by traditional surveys, although the traditional suveys should be combined to verify the results. We revealed that there is high species richness of Cypriniformes in the lower reaches and benthic species are important across the river system. This is important information about fish communities along the Ishikari River system on the Kamikawa Plain and the conservation of the aquatic ecosystems of Hokkaido Island.

## Supplementary Material

B7CB6857-9B56-53F1-8FE8-2291AE4EA2FD 10.3897/BDJ.8.e56876.suppl1 Supplementary material 1 Table S1 Results of eDNA metabarcoding for 16 sites from October 2018 to August 2019. Data type xlsx spreadsheetxlsx spreadsheet Brief description Results of eDNA metabarcoding for 16 sites from October 2018 to August 2019. Total number of species per site is shown numerically and the presence/absence is indicated by 1/0, respectively. Site abbreviations correspond to those in Figures. File: oo_458807.xlsx https://binary.pensoft.net/file/458807 A. Imamura, K. Hayami, M.K. Sakata, T. MinamotoA. Imamura, K. Hayami, M.K. Sakata, T. Minamoto

39663944-8E81-5784-AA2B-F7A7B2C515D0 10.3897/BDJ.8.e56876.suppl2 Supplementary material 2 Raw data of eDNA metabarcoding. Data type Spread sheet Brief description Marine fish species are omitted. Species identification is tentative. File: oo_458806.xlsx https://binary.pensoft.net/file/458806 A. Imamura, K. Hayami, M. K. Sakata, T Minamoto

2C8A8F90-5B40-582A-BFB6-1CD960339BD8 10.3897/BDJ.8.e56876.suppl3 Supplementary material 3 Table S2 Data type xlsx spreadsheets Brief description Results of fish species detected. File: oo_458724.xlsx https://binary.pensoft.net/file/458724 A. Imamura, K. Hayami, M. K. Sakata, T Minamoto

05D4E492-E57C-5721-BAFA-6D8BF768128B 10.3897/BDJ.8.e56876.suppl4 Supplementary material 4 Supplementary figures of the environmental DNA survey Data type jpeg images Brief description Fig. S1 Results of the environmental DNA survey for each fish species. Circle size indicates the total number of samples in which that species was detected out of the 176 samples taken throughout the year (October 2018–August 2019). The explanatory circle with “10” indicates that the species was detected in ten samples. SI_a: *Lethenteron* sp.; SI_b: *Tanakia lanceolata* (slender bitterling); SI_c: *Rhynchocypris lagowskii* (Amur minnow); SI_d: *Gnathopogon caerulescens* (hon-moroko); SI_e: *Silurus asotus* (Far Eastern catfish); SI_f: *Pungitius* sp.; SI_g: *Oryzias* sp.; SI_i: *Rhinogobius* sp. File: oo_458729.pdf https://binary.pensoft.net/file/458729 A. imamura, K. Hayami, M. K. Sakata, T. Minamoto

8EAB09A3-8A1A-504D-BBC4-4E0BC40CE901 10.3897/BDJ.8.e56876.suppl5 Supplementary material 5 Table S3 Result of Permutational Multivariate Analysis of Variance (PerMANOVA) for the fish community detected with eDNA metabarcoding. The sites were categorised as mainstream, mid-size stream or small tributary Data type xlsx spreadsheet File: oo_445872.xlsx https://binary.pensoft.net/file/445872 A. Imamura, K. Hayami, M.K. Sakata, T. Minamoto

7C507588-3915-5CA1-A9DE-41055161E40D 10.3897/BDJ.8.e56876.suppl6 Supplementary material 6 Supplementary Tables S4-S10 Data type xlsx spreadsheets Brief description Result of Generalised Linear Model (GLM) for the detection of major fish species. File: oo_458725.xlsx https://binary.pensoft.net/file/458725 A. Imamura, K. Hayami, M.K. Sakata, T. Minamoto

## Figures and Tables

**Figure 1.**
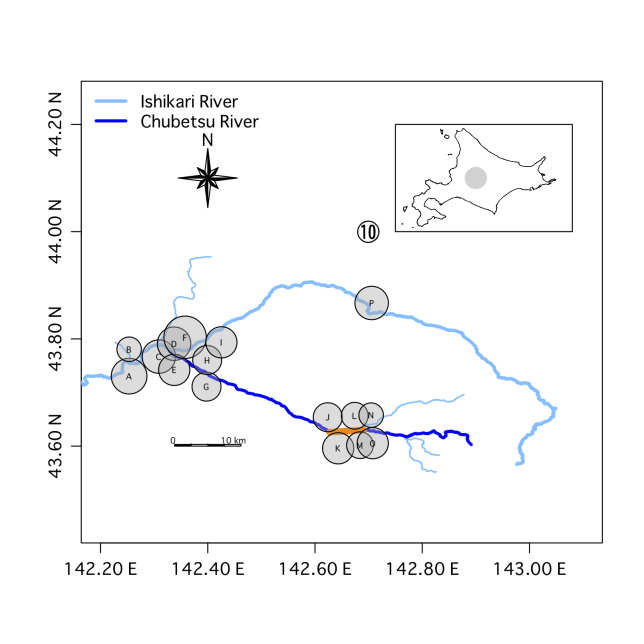
Results of the environmental DNA metabarcoding of 176 samples from 16 sites in the Ishikari River system. Circle size indicates the total number of species detected over 12 months (October 2018 to August 2019). There were 11–20 species per site. The explanatory circle with a “10” indicates the circle size for a site in which ten species were detected. The letters denote each site as described in Suppl. material 1 (the same letters are used in Figs 2, 3, 4 and Suppl. material 4). The orange line indicates the Chubetsu Reservoir and the grey circle in the inset map indicates the region of the research sites on Hokkaido Island.

**Figure 2a.**
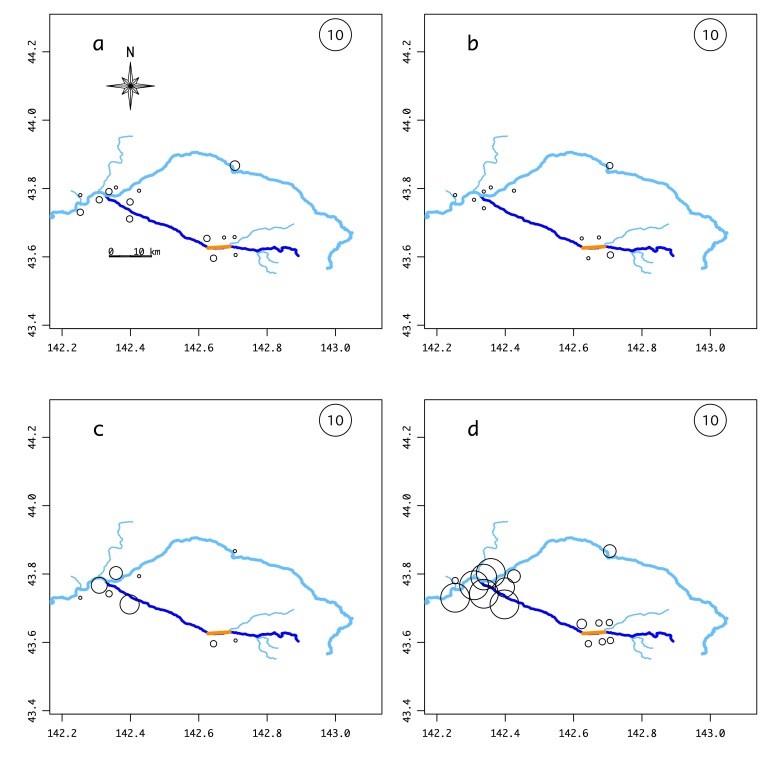


**Figure 2b.**
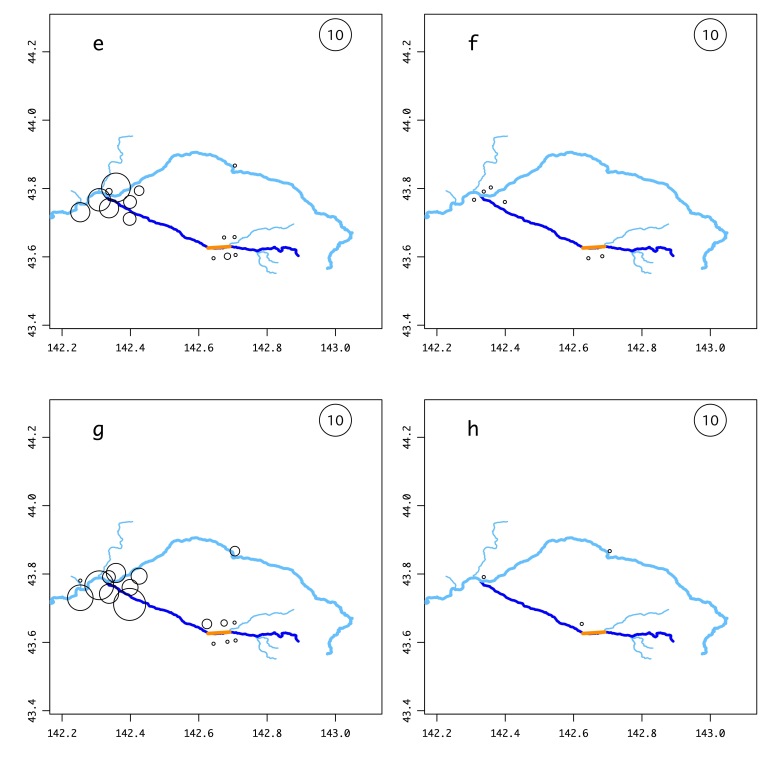


**Figure 2c.**
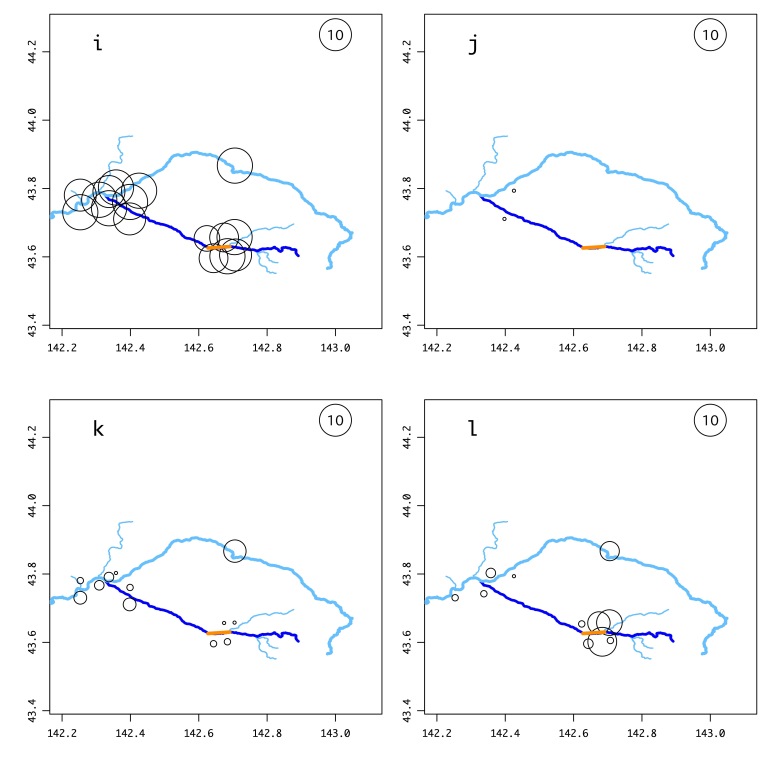


**Figure 2d.**
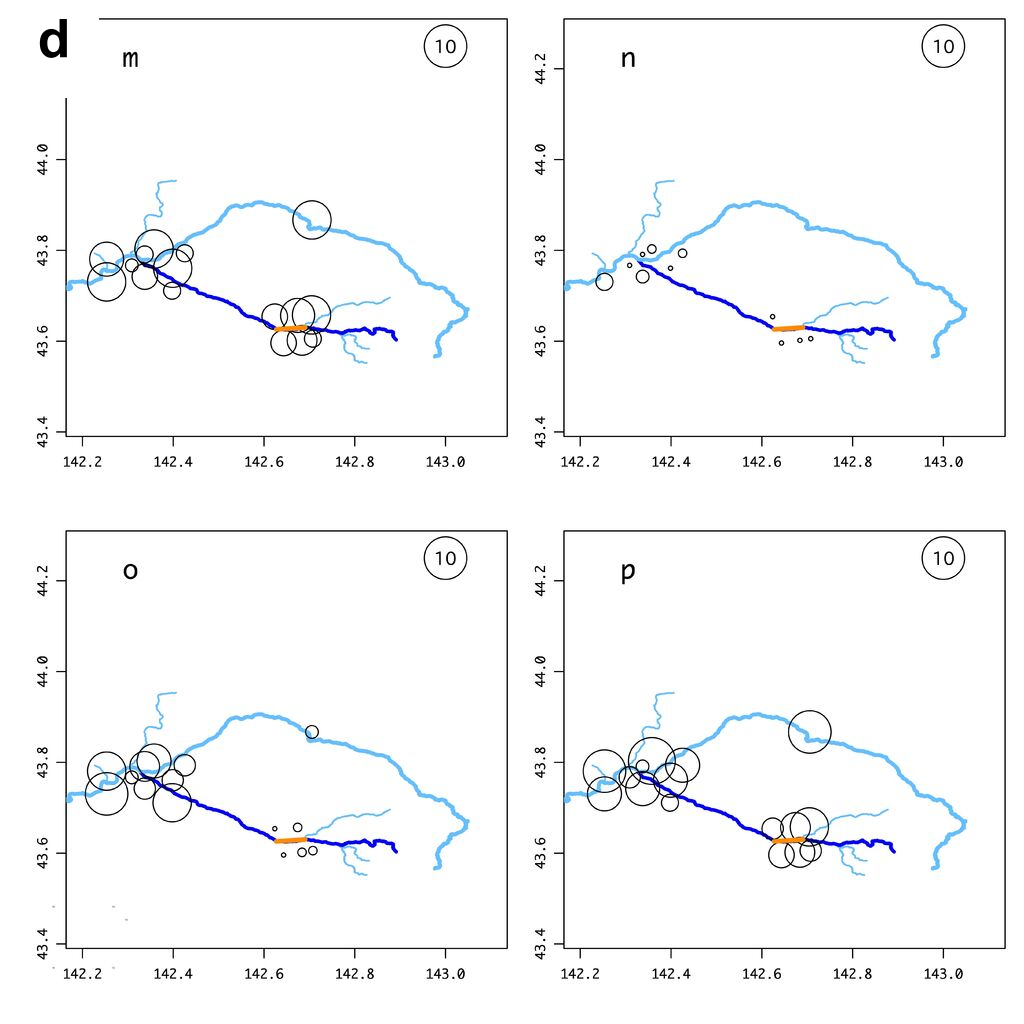


**Figure 3.**
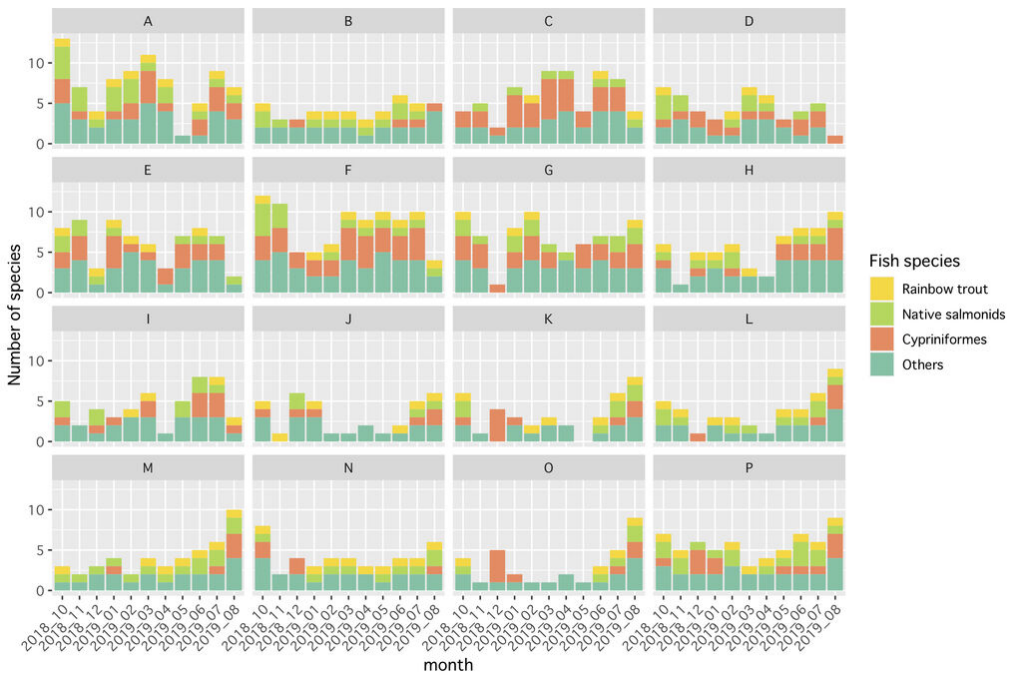
Seasonal change in species detected by eDNA metabarcoding at each site. Species were classified into the following groups: invasive *Oncorhynchus mykiss* (rainbow trout), native salmonids, Cypriniformes and others.

**Figure 4.**
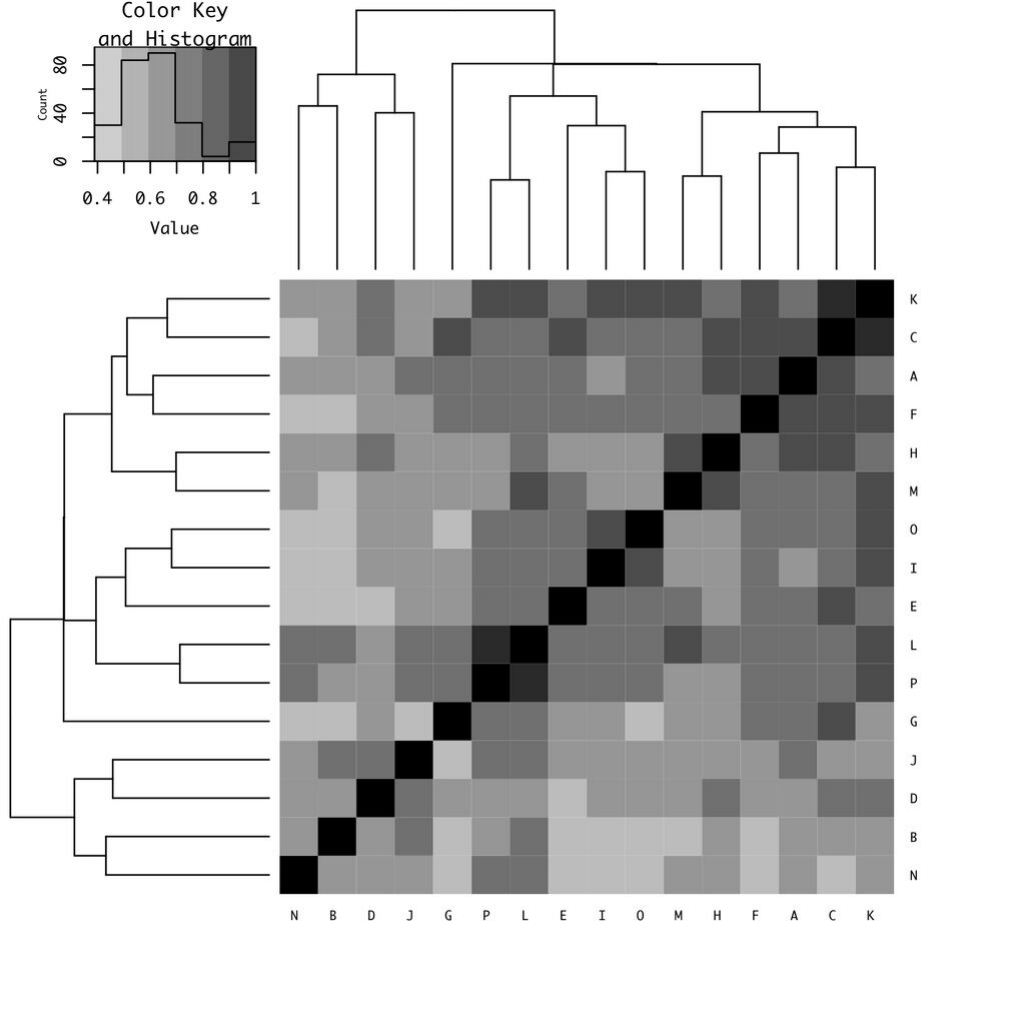
The similarity of the fish communities between sites drawn as a heatmap. The distance matrix using the Jaccard Index was calculated and clustering was executed with “average” in the R package “gplot.” The histogram indicates the mode of the similarity (0.60) and the solid line is the middle of max and min of the similarity (about 0.69).
