# Supplementary figures and images for: Environmental DNA revealed the fish community of Hokkaido Island, Japan, after invasion by rainbow trout
Source: Biodivers Data J. 2020 Oct 29;8:e56876. doi: 10.3897/BDJ.8.e56876 (PMC7644654; doi:10.3897/BDJ.8.e56876)

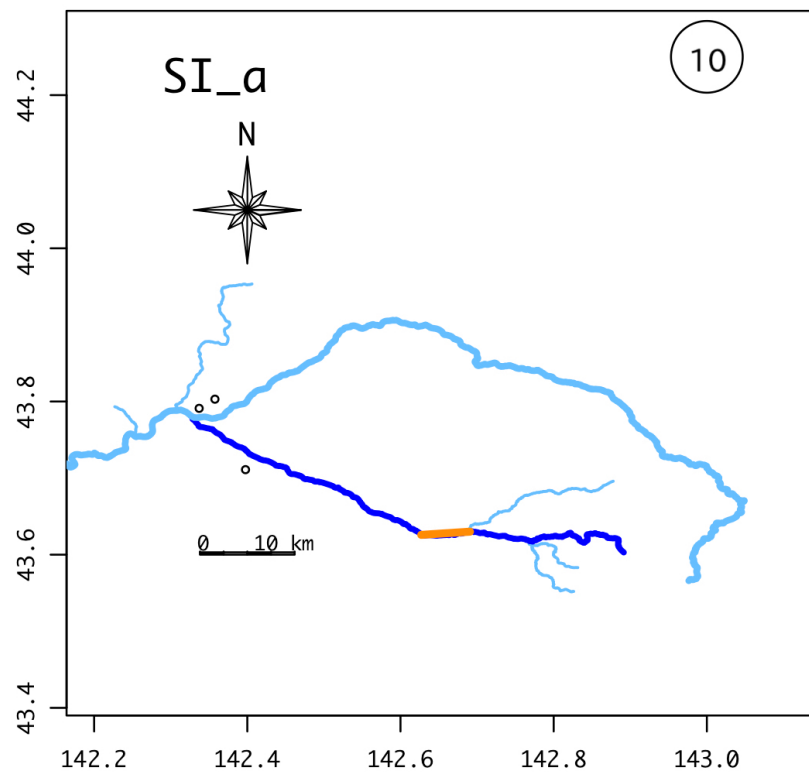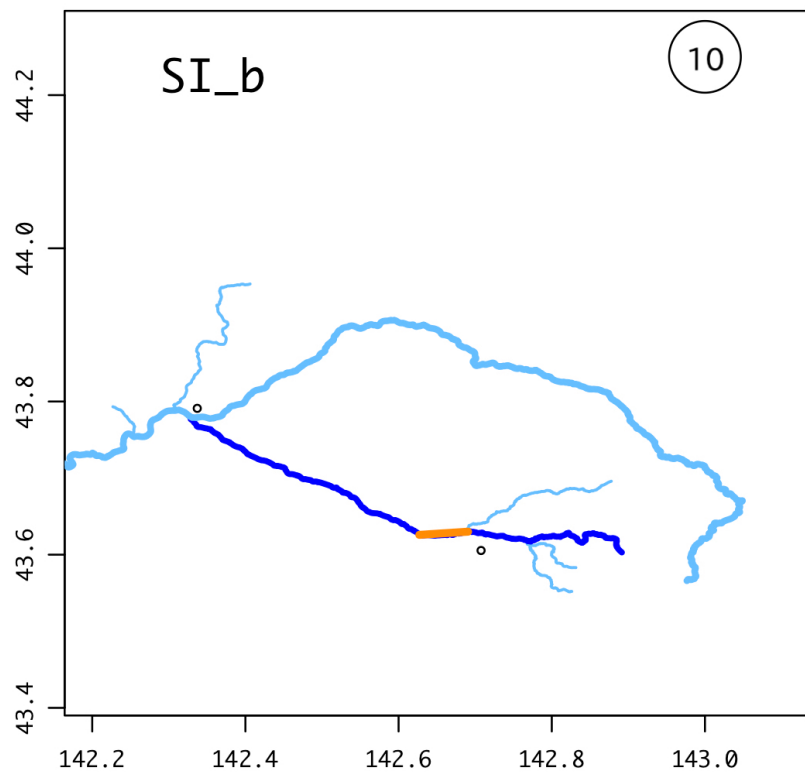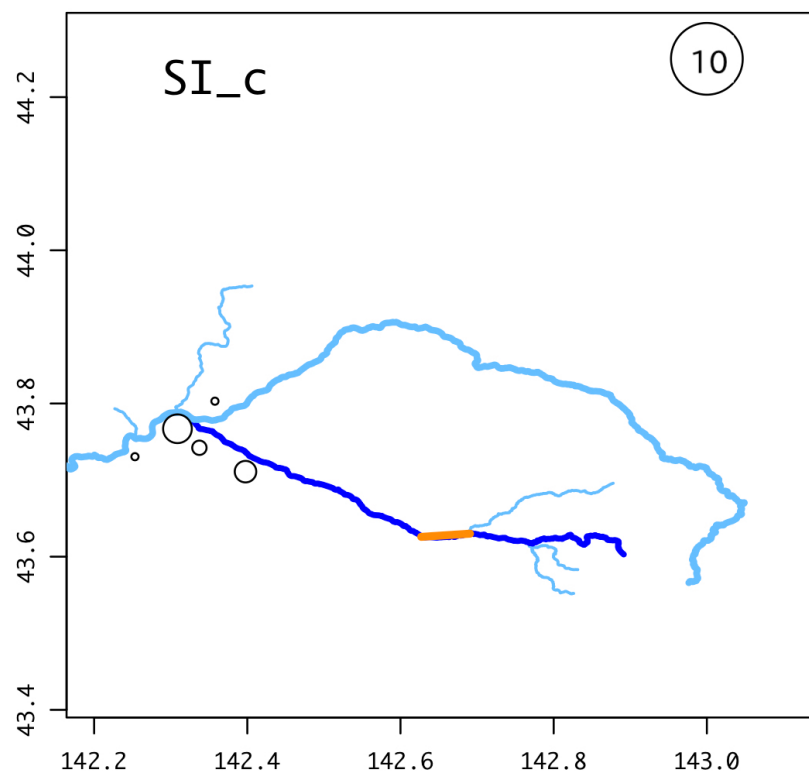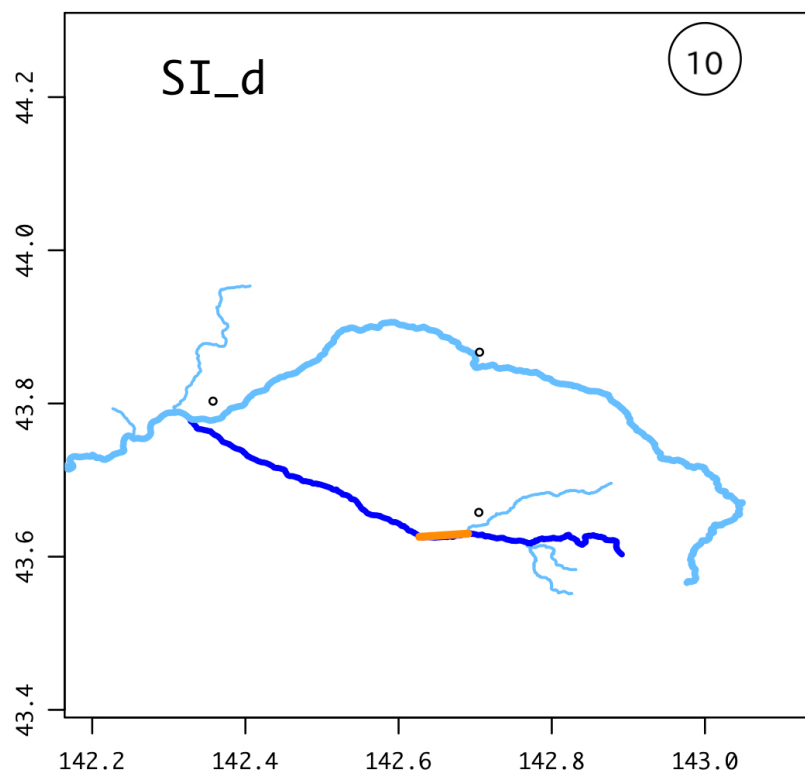

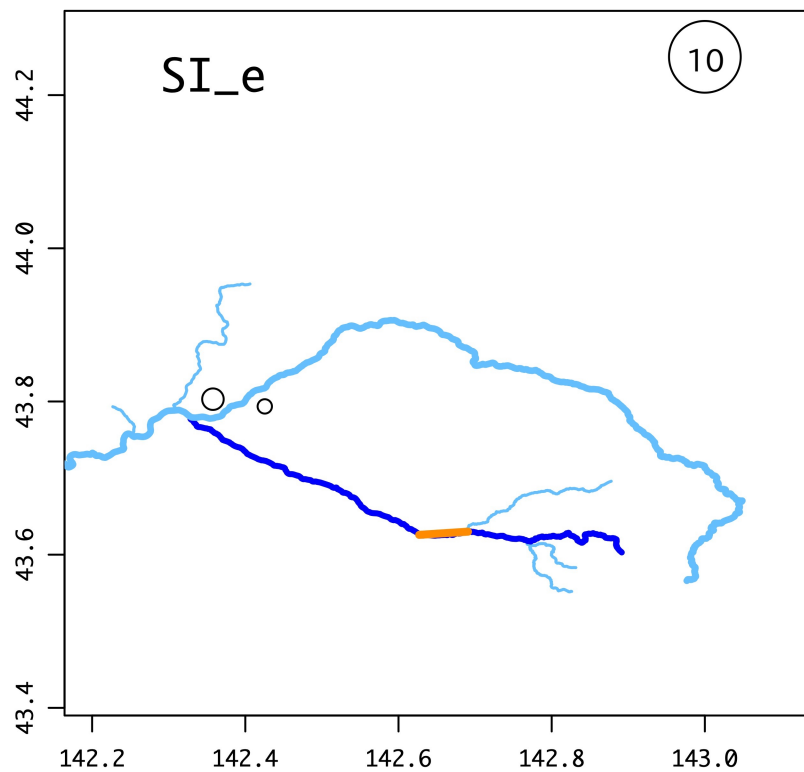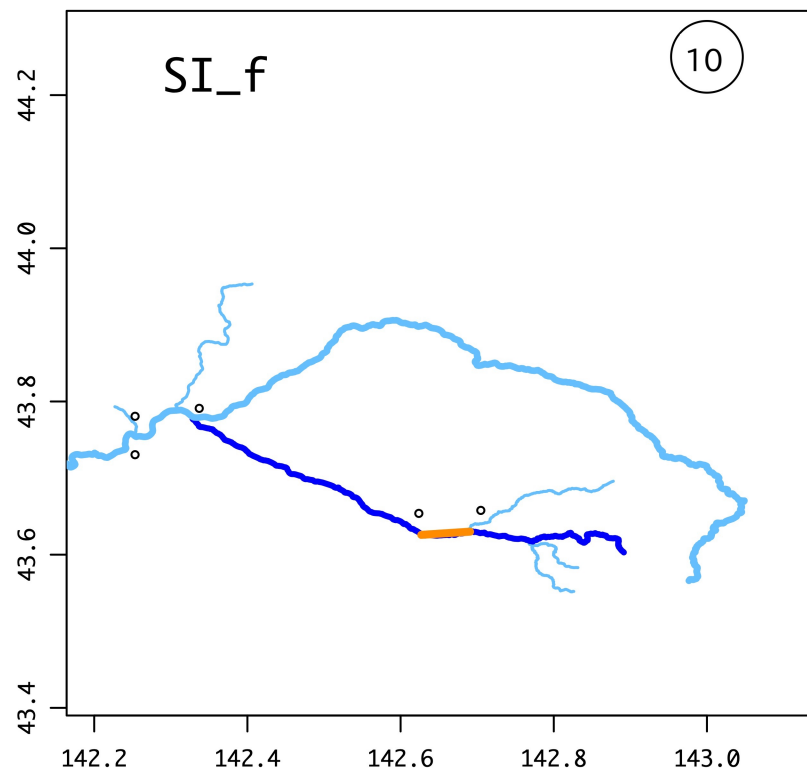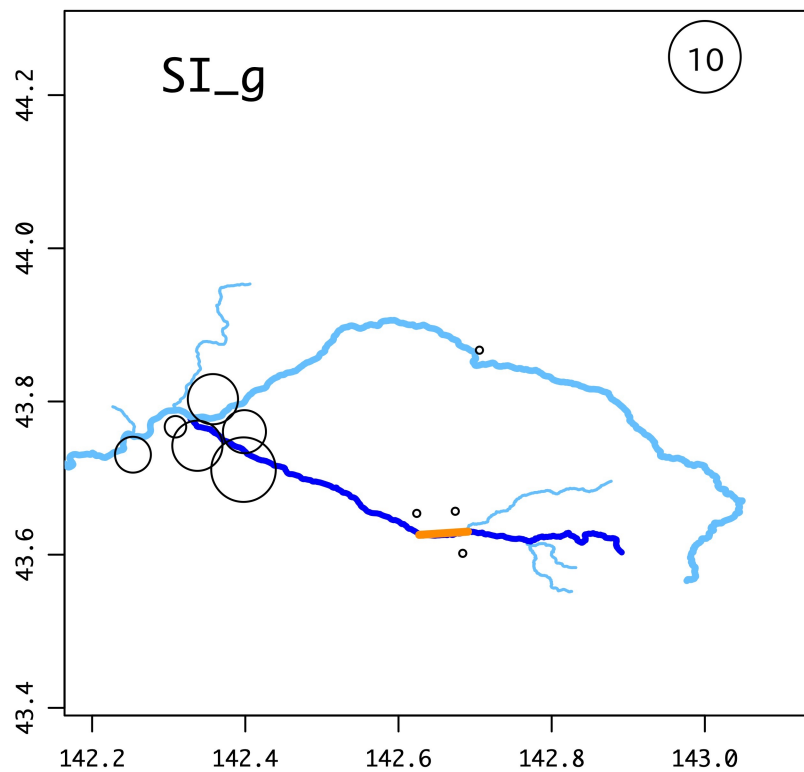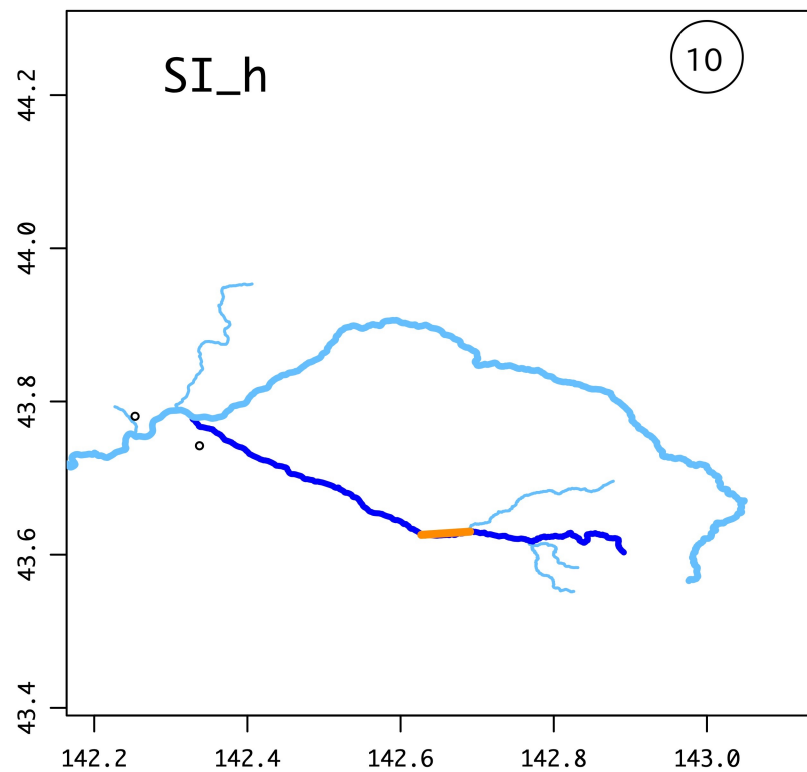

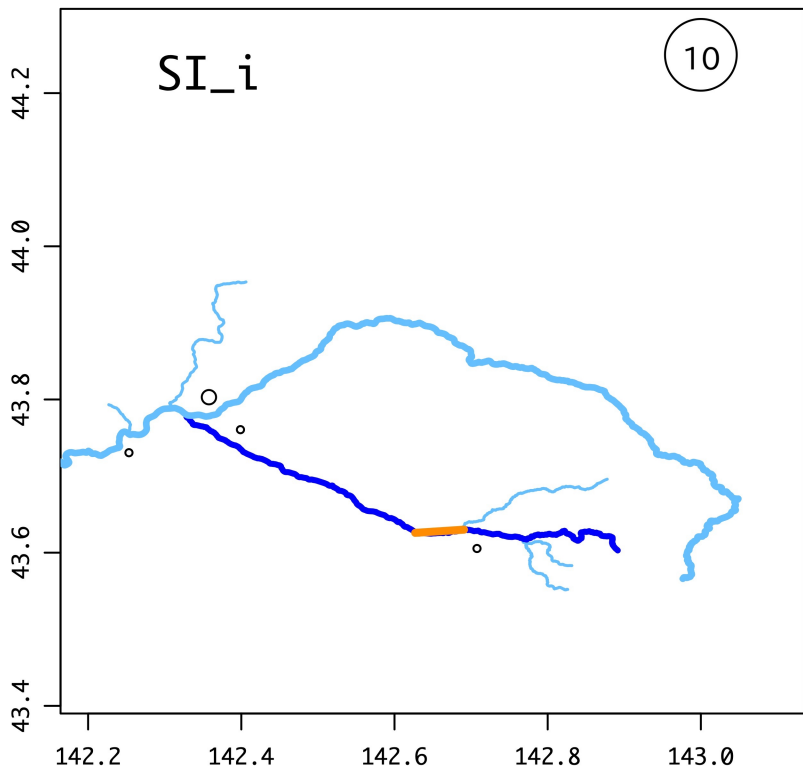

Supplement: Supplementary material 4 — Supplementary figures of the environmental DNA survey [file bdj-08-e56876-s004.pdf]
